# Leveraging Context for Perceptual Prediction Using Word Embeddings

**DOI:** 10.1111/cogs.70072

**Published:** 2025-06-07

**Authors:** Georgia‐Ann Carter, Frank Keller, Paul Hoffman

**Affiliations:** ^1^ Institute for Language Cognition and Computation, School of Informatics The University of Edinburgh; ^2^ School of Philosophy Psychology and Language Sciences The University of Edinburgh

**Keywords:** Word embeddings, Perceptual information, Conceptual combinations, Neural networks

## Abstract

Word embeddings derived from large language corpora have been successfully used in cognitive science and artificial intelligence to represent linguistic meaning. However, there is continued debate as to how well they encode useful information about the perceptual qualities of concepts. This debate is critical to identifying the scope of embodiment in human semantics. If perceptual object properties can be inferred from word embeddings derived from language alone, this suggests that language provides a useful adjunct to direct perceptual experience for acquiring this kind of conceptual knowledge. Previous research has shown mixed performance when embeddings are used to predict perceptual qualities. Here, we tested if we could improve performance by leveraging the ability of Transformer‐based language models to represent word meaning in context. To this end, we conducted two experiments. Our first experiment investigated noun representations. We generated decontextualized (“charcoal”) and contextualized (“the brightness of charcoal”) Word2Vec and BERT embeddings for a large set of concepts and compared their ability to predict human ratings of the concepts’ brightness. We repeated this procedure to also probe for the shape of those concepts. In general, we found very good prediction performance for shape, and a more modest performance for brightness. The addition of context did not improve perceptual prediction performance. In Experiment 2, we investigated representations of adjective–noun phrases. Perceptual prediction performance was generally found to be good, with the nonadditive nature of adjective brightness reflected in the word embeddings. We also found that the addition of context had a limited impact on how well perceptual features could be predicted. We frame these results against current work on the interpretability of language models and debates surrounding embodiment in human conceptual processing.

## 1 Introduction

Our understanding of the world is shaped by the perceptual information we take in through our experiences (Gibbs Jr, 2005; Rogers & Wolmetz, 2016). This perceptual information can be important conceptually—for example, we know that dark chocolate tastes more bitter than white chocolate. The degree to which such perceptual information shapes semantic representations has been a core debate within the cognitive sciences (Barsalou, 2008; Louwerse, 2011; Pylyshyn, 1980; Rogers & McClelland, 2004). One key question that has emerged from this concerns how much experiential information can be learnt from linguistic content alone (Dove, 2014). Computational models trained on large language corpora provide an important perspective on this debate. These models appear to derive sophisticated semantic representations from linguistic input alone, with no access to perceptual experience. Language models typically generate high‐dimensional representations for words and phrases termed “embeddings,” which situate concepts in a semantic space. However, a core criticism of these word embeddings is that their dimensions are difficult to interpret and the degree to which they faithfully represent the perceptual aspects of semantics (like the color of chocolate) remains uncertain (Chersoni, Santus, Huang, & Lenci, 2021; Ettinger, 2020). One way to tackle this issue is to compare word embeddings with human ratings or neuroimaging data to examine the extent to which they mimic human semantics (Abnar, Ahmed, Mijnheer, & Zuidema, 2018; Ettinger, 2020; Hollenstein, de la Torre, Langer, & Zhang, 2019; Turton, Vinson, & Smith, 2021, 2020; Utsumi, 2020). In this vein, the present study aims to test how well model‐derived embeddings predict human judgments of the salience of specific perceptual properties of objects, and whether additional linguistic context improves these predictions. In so doing, we hope to provide new insights into the degree to which perceptual knowledge can be acquired from language alone.

### 1.1 Conceptual processing: Theories and frameworks

Historically, two opposing theoretical perspectives on the nature of semantic representations have been contrasted. The symbolic account of cognition claims that meaning is extracted from language and abstracted into amodal representations (Pylyshyn, 1980). In contrast, the embodied cognition account proposes that human cognition (and by extension, language) is fundamentally grounded in sensorimotor experiences and systems (Bolognesi & Steen, 2018; Gibbs Jr, 2005). Between these extremes, a number of hybrid perspectives envision roles for both language‐derived representations and perceptual grounding in supporting semantic processing (Andrews, Frank, & Vigliocco, 2014; Barsalou, Santos, Simmons, & Wilson, 2008; Louwerse, 2018). Among these accounts, the nature of the representations themselves, as well as the extent to which perceptual information is vital for the formation of these representations, is under debate (Kiefer & Pulvermüller, 2012). Symbolic and embodied perspectives have been supported by an extensive amount of research that has often fallen along different methodological lines, with much of the evidence for symbolic cognition coming from computational models, while the evidence for embodied cognition can be found in studies with human experiments (Andrews et al., 2014; Louwerse, 2018).

There is now widespread evidence from both behavioral and neuroimaging experiments that supports the idea that perceptual representations are often activated during language comprehension (Hauk, 2016; Kiefer & Pulvermüller, 2012; Louwerse, 2018; Meteyard, Cuadrado, Bahrami, & Vigliocco, 2012). For example, response latencies from picture verification tasks have demonstrated that comprehenders are sensitive to the orientation of objects in an image, as well as the shape of objects (Stanfield & Zwaan, 2001; Zwaan, Stanfield, & Yaxley, 2002). Here, response latencies were shorter for pictures of objects that matched in orientation or shape with that suggested by prior context, than for pictures of objects that did not match. Meanwhile, evidence from neuroimaging has provided additional insights into the between semantic and perceptual information. For example, Simmons and colleagues (2007) demonstrated that the region of cortex strongly linked to color perception is also active when processing color terms presented as the properties of objects (e.g., “GRASS‐green”). Similar neuroimaging results have also been obtained for other perceptual modalities, such as action (Hauk, Johnsrude, & Pulvermüller, 2004).

However, much of this research has traditionally treated conceptual representations as static and context‐free, without addressing the flexibility that occurs when concepts are used in different contexts (Hoffman, Lambon Ralph, & Rogers, 2013; Yee & Thompson‐Schill, 2016). Many words have radically different connotations in different situations. The word “bank” should evoke the percept of a large building when used in the context of a city street, but that of a grassy slope when used in the context of a river. Task also influences embodiment: the same words can engage either the visual system or the motor system, depending on which properties are relevant to the participant's current task (van Dam, van Dijk, Bekkering, & Rueschemeyer, 2012). Findings like these suggest that people flexibly reshape their semantic representations as they encounter different situations (Barsalou, 1983; Jamieson, Johns, Vokey, & Jones, 2022). They also suggest that the degree to which perceptual information is activated depends on the task we are performing and the context in which that word is presented (Barsalou et al., 2008).

To understand the degree to which perceptual information is embedded in language, many researchers have investigated the capabilities of computational language models that are solely exposed to language input. We review these findings in the next section. For now, it is important to highlight that much of this research has also been conducted from a context‐free perspective. Pioneering models like latent semantic analysis (LSA; Landauer & Dumais, 1997), as well as more recent models such as word2vec (Mikolov, Chen, Corrado, & Dean, 2013; Mikolov, Sutskever, Chen, Corrado, & Dean, 2013) and GloVe (Pennington, Socher, & Manning, 2014) have been invaluable in discovering the semantic structure present in language. But these models represent each word as a context‐independent, static embedding. Given the importance of context in shaping human semantic representation, these context‐free representations likely underestimate the semantic information present in language. More recent developments in natural language processing, in the form of Transformer‐based models, allow contextualized word embeddings to be generated (Devlin, Chang, Lee, & Toutanova, 2018; Misra, Ettinger, & Rayz, 2020; Ontanon, Ainslie, Fisher, & Cvicek, 2022). These embeddings provide a contextualized representation of a word, such that the embeddings for a polysemous word, such as “bank,” will be different across sentences that make use of the distinct senses of the word. In this study, we investigate the types of perceptual information present in different forms of contextualized embeddings.

### 1.2 Interpreting the semantic content of word embeddings

Word embeddings, as vector‐based representations of language, are strongly related to the distributional hypothesis. This is the notion that the semantic similarity between two linguistic expressions can be understood as a function of the similarity of the linguistic contexts that they appear in (Firth, 1957; Harris, 1954; Lenci, 2008). This has widely influenced the cognitive science of semantics, where the distributional hypothesis is also proposed as a cognitive hypothesis for the organization of meaning. As such, models derived from distributional language data have been used to model multiple aspects of human language processing, such as word association, semantic deficits, and categorization (Bullinaria & Levy, 2007; Griffiths, Steyvers, & Tenenbaum, 2007; Vigliocco, Vinson, Lewis, & Garrett, 2004). Early examples of these distributional models include LSA and the Topic model (Griffiths et al., 2007; Landauer & Dumais, 1997), while more recent examples include Word2Vec and GloVe (Mikolov et al., 2013; Mikolov et al., 2013; Pennington et al., 2014).

Word embeddings extracted from these models can be represented geometrically in a semantic space, where words that are more semantically related cluster together (Riordan & Jones, 2011). Because of this, semantic similarity has commonly been used as an evaluation metric for distributional representations (Günther, Dudschig, & Kaup, 2016; Jones, Kintsch, & Mewhort, 2006; Lenci, 2008; Lowe & McDonald, 2000). For example, Grand and colleagues used semantic projection to compare the internal representations of word embeddings against human judgments of object categories and properties. The authors constructed scales denoting a semantic feature of interest, say size, and were able to compare the internal representations of word embeddings by creating a featural subspace where size influences the similarity patterns between the embeddings. They found that these feature‐wise similarities predicted human feature judgments, concluding that the geometric representation of word embeddings contains rich conceptual knowledge (Grand, Blank, Pereira, & Fedorenko, 2022).

Another approach has focused on learning a mapping between word embeddings and property norms as a way to ground them in interpretable representations (Chersoni et al., 2021; Derby, Miller, & Devereux, 2019; Fǎgǎrǎşan, Vecchi, & Clark, 2015; Utsumi, 2020). Previous research using this approach has produced mixed findings on the degree to which word embeddings mimic perceptual aspects of human semantics. Abdou and colleagues (2021) explored BERT, RoBERTa, and ELECTRA embeddings for color words (e.g., “yellow”) using representational similarity analysis (RSA) and linear regression. They found that the embeddings of color words aligned with the structure of a 3D color space, CIELAB, concluding that an approximation of the perceptual color space can be extracted from text alone. However, this success in extracting color knowledge from embeddings has not been replicated when probing for color information about objects (e.g., bananas are yellow). Sommerauer and Fokkens (2018) used Word2Vec embeddings to classify objects according to whether they possessed particular features (e.g., is yellow, is dangerous). While functional and behavior‐relevant features were generally classified well, performance was poor for perceptual features, including color. In a similar vein, Lucy and Gauthier (2017) evaluated word embeddings on how well they predicted perceptual and conceptual features of concrete concepts, using semantic norm datasets collected from humans as a gold‐standard. They tested different types of word embeddings (GloVe and Word2Vec) using the McRae and CSLB semantic norm datasets (Devereux, Tyler, Geertzen, & Randall, 2014; McRae, Cree, Seidenberg, & McNorgan, 2005) and found that the embeddings failed to encode many salient perceptual features of the concepts, in comparison to strictly nonperceptual categories (such as taxonomic and functional features).

The previously mentioned studies used embeddings to predict the presence or absence of binary features (e.g., is yellow vs. is not yellow). Meanwhile, other studies have tried to predict continuous ratings of the importance or relevance of different types of information. For example, Chersoni and colleagues (2021) trained a neural network to learn the mapping from word embeddings (both count‐based models, e.g., PPMI, GloVe, and prediction‐based models, e.g., SGNS, BERT) to vectors devised from human ratings. The human‐based vectors were taken from the Binder dataset (Binder et al., 2016), which contains ratings on the relevance of 65 semantic features to 535 concepts. Participants were asked to rate the relevance of each semantic feature for a particular concept. The 65 features were selected to represent core modalities of information processing from the neuroimaging literature. For example, the dataset includes features focusing on specific sensory and motor experiences (e.g., shape and motion), as well as affective experiences (e.g., happy and sad). The models were tested on their ability to predict values over all 65 features for unseen words. The authors found that social, causal, and cognition features were generally better predicted than sensorimotor features, consistent with the idea that language is a critical source of information for these types of semantic features (Borghi et al., 2019). Within the perceptual domain, some somatosensorial features were well‐predicted (such as color and shape), whereas others were less well captured (such as bright and dark) (Chersoni et al., 2021).

A number of other studies have used the Binder dataset to investigate the knowledge represented in word embeddings. Turton and colleagues (2021, 2020) used both Word2Vec and BERT embeddings to predict the feature rating vectors in the Binder dataset, finding that some perceptual features were again well predicted (such as color and shape), but others were less well represented (such as bright, dark, and slow). They also demonstrated that the mappings learnt between word embeddings and the feature rating vectors can be extrapolated to a wider vocabulary than the original dataset, while keeping the semantic relationships between features intact. Utsumi (2020) conducted a similar experiment evaluating the mappings between the feature rating vectors from the Binder dataset and word embeddings using three types of distributional embeddings (SGNS, GloVe, and PPMI) that were derived from training on two different corpora (COCA and Wikipedia). Similar to Chersoni et al. (2021), they found that social, causal, and cognition features were better predicted, with perceptual features less likely to be represented in word embeddings. For example, features relating to the brightness or speed of a concept were predicted poorly. However, Utsumi concluded that some ability to predict perceptual information was present for domains such as shape, vision, and sound. Utsumi (2020) also investigated the prediction performance separately among concrete and abstract concepts. Predictions were poorer for abstract words across all feature types, except for emotion features. Perhaps unsurprisingly, perceptual features were predicted particularly poorly for abstract words, consistent with the long‐standing view that abstract concepts have few perceptual associations (Paivio, 1990). Taken together, these studies provide some evidence that embeddings are able to predict some types of perceptual information associated with concepts, though prediction of purely perceptual features is poorer than for other types of semantic information.

Why do perceptual qualities seem to be less well‐represented in word embeddings? In the previous section, we noted that the engagement of perceptual processing during language comprehension is highly dependent on context. However, traditional distributional methods for obtaining word embeddings (e.g., Word2Vec, GloVe) have been decontextualized (also known as “static”). This means that the entire word representation is encoded as a single vector, abstracted across all the different contexts in which the word is used. As such, static embeddings capture the most significant semantic properties and relationships that are most reliably represented across contexts. This means that less salient information, such as perceptual qualities, may not be well‐represented. However, further advances in machine learning have led to Transformer‐based LLMs, where a word's embedding changes depending on the linguistic context in which it is presented (Vaswani et al., 2017). These models have more sophisticated embeddings with the potential to encode context‐specific information, such as different word senses and contextually relevant properties. Researchers have begun to test the predictive abilities of contextualized word embeddings using the Transformer‐based BERT model (Devlin et al., 2018). Turton, Smith, and Vinson (2021) generated BERT embeddings for concepts by sampling 250 sentences containing each word in the Binder dataset. These sentences were input into BERT, providing 250 different contextualized word embeddings for each target word. A single, context‐free representation was then obtained for each word by averaging over its 250 embeddings (for similar approaches, see Chersoni et al., 2021; Bommasani, Davis, & Cardie, 2020; Vulić, Ponti, Litschko, Glavaš, & Korhonen, 2020). Turton et al. (2021) found that BERT embeddings created in this way outperformed static embeddings in predicting the feature rating vectors from Binder et al. (2016), suggesting that simply aggregating embeddings over many contexts leads to representations that better capture human experience with concepts. They went on to demonstrate that handpicking 10 sentences which matched the sense of the word as used in the Binder dataset improved this result further.

The benefit of contextualized embeddings was also demonstrated when assessing the effects of specific contexts. Here, Turton and colleagues (2021) used a dataset of semantic feature ratings for property–object pairs (e.g., “abrasive lava”; “abrasive sandpaper”; Van Dantzig, Cowell, Zeelenberg, & Pecher, 2011) to investigate how the presence of a specific context influences prediction performance. In the original study, participants were asked to provide ratings on five separate scales representing each perceptual modality in answer to the prompt: “To what extent do you experience [object] being [property]” (Van Dantzig et al., 2011). Turton and colleagues first fed property–object pairs into the Transformer models and extracted the word embeddings for the property words in the context of a specific object. They then compared the performance of these embeddings against an averaged property embedding (i.e., averaged across two object contexts) and a static baseline Numberbatch embedding in predicting feature ratings for the property–object pairs (Speer, Chin, & Havasi, 2017). For example, they compared how well the extracted embeddings for “abrasive” predicted the perceptual feature ratings for “abrasive lava” and “abrasive sandpaper.” They found that the contextualized Transformer embeddings outperformed the mean Transformer embeddings and the Numberbatch embeddings. This study provided a first indication that contextualizing embeddings with a specific use case can lead to more effective prediction of perceptual properties. In the present study, we build on this idea in two experiments. In the first, we investigate whether the prediction of human‐generated ratings on the perceptual properties of nouns is improved when contextualized embeddings are generated using a context that specifically primes for the desired perceptual feature (e.g., “the brightness of charcoal,” “the shape of charcoal”). We investigated both ratings on the relevance of the feature to the noun (either brightness or shape), and ratings on the perceived brightness of the noun. In the second experiment, we extend this to adjective–noun phrases (e.g., “dark charcoal”), which have rarely been studied. Here, we attempt to predict ratings of the perceived brightness of conceptual combinations. We tested whether contextualized embeddings better predict the perceptual ratings of such phrases and whether language models compose the meanings of adjective–noun phrases in a similar way to humans.

## 2 Experiment 1

In Experiment 1, we compared contextualized and decontextualized word embedding performance on how well they can predict human‐generated ratings of the perceptual qualities of nouns. We explored the prediction performance of word embeddings from a Distributional Semantic Model (Word2Vec) and a Large Language Model (BERT). We investigated these issues using two specific perceptual features as test cases: brightness and shape. We chose brightness as it has not been predicted well in previous research (Chersoni et al., 2021; Utsumi, 2020), and, therefore, represents a challenging test case for examining the extent of perceptual feature representation in embeddings. This is in contrast to shape, which has been well‐predicted (Chersoni et al., 2021; Turton et al., 2021; Utsumi, 2020). We also selected brightness because it allowed us to make use of a critical dataset that contained ratings for both unmodified nouns, and adjective–noun combinations (the Solomon and Thompson‐Schill dataset), which allowed us to explore the nature of conceptual combinations in context.

First, we used bright and dark ratings from the Binder dataset, which has been most commonly used in previous research on this topic (Chersoni et al., 2021; Turton et al., 2021, 2020; Utsumi, 2020). Binder et al. (2016) obtained ratings for many different features so this dataset contains a large number of items for which brightness is not a salient (e.g., “chair”) or relevant feature (e.g., “advantage”). Second, we used the brightness dataset of Solomon and Thompson‐Schill (2020). S&T‐S only explored brightness and, therefore, they collected ratings for a smaller set of concepts for which brightness/darkness was a relevant and salient feature (e.g., “diamond,” “gray,” “charcoal”). By comparing perceptual prediction across these two datasets, we were able to test the degree to which findings from the Binder dataset generalize to other datasets, which are tailored to the specific feature under investigation. For comparison, we also predicted ratings on the relevance of shape to the concepts. We selected this perceptual feature as it has previously been reported to be well predicted by BERT embeddings (Chersoni et al., 2021; Turton et al., 2020). We used the Binder ratings for this investigation.

**Fig. 1:**
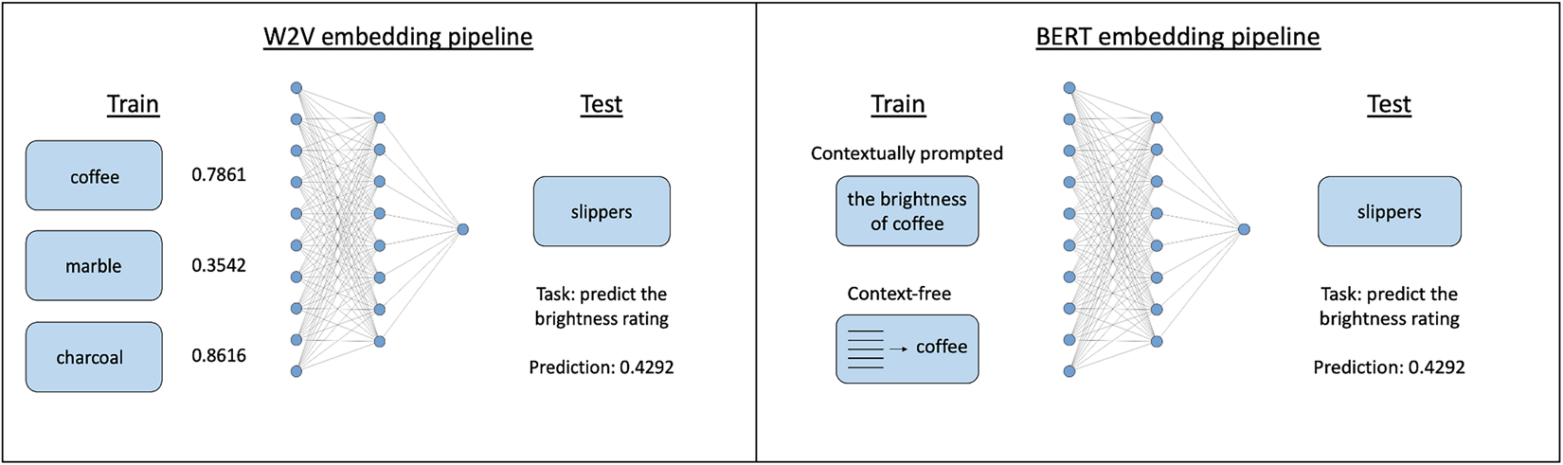
Experiment 1 pipelines.

Fig. 1 shows an overview of our experiment pipelines. For the Word2Vec embeddings, we extracted embedding representations of the nouns from Google's pretrained model that was trained on a section of the Google News dataset (Mikolov et al., 2013; Mikolov et al., 2013). We then used these embeddings as input to a feed‐forward neural network to predict the perceptual feature ratings for that noun. Word2Vec generates a single representation of each word and, therefore, these embeddings are decontextualized and static. In contrast, we used BERT's capacity for contextualization when extracting our BERT embeddings. Here, we make use of Google's pretrained models, extracting embeddings from both the BERT base model and the BERT large model, which differ in dimensionality (Devlin et al., 2018). Following previous studies, we included a context‐free condition, which contained an averaged BERT embedding from multiple sentence contexts (Bommasani et al., 2020; Chersoni et al., 2021; Turton et al., 2021; Vulić et al., 2020). This context‐free condition can also be thought of as a prototype because it derives an abstract representation of each word across many different instances (Hampton, 2015; Rosch & Mervis, 1975). To do this, we identified 250 sentences that contained the noun of interest from the one Billion Words Benchmark corpus (Chelba et al., 2014). We then extracted the BERT embedding for the noun in each sentence and averaged these. We also had a contextually prompted condition, where we included a contextual prompt targeted toward the perceptual feature of interest. For our brightness investigations, we made use of two different prompts, “the brightness of…” and “the color of…”; while we only used one prompt for the shape investigations: “the shape of….” We separately presented these prompts to BERT and extracted the embedding for the noun. If the contextualization of BERT embeddings produces embeddings that align with human judgments of conceptual flexibility, we would expect the contextually prompted BERT embedding to better predict the perceptual feature of interest, compared to both the Word2Vec and context‐free BERT embeddings.

### 2.1 Methods

All associated code and data can be accessed here: https://osf.io/ca4wm/

#### 2.1.1 Datasets

We evaluated model performance on predicting human ratings of perceptual qualities from two datasets. In the *Solomon and Thompson‐Schill (S&T‐S) dataset*,^1^ Solomon and Thompson‐Schill (2020) asked participants to rate the brightness of 45 nouns on a scale of 0–50 (brightest to darkest), both with and without modifiers. Participants (*n* = 100) made these judgments by moving a slider, with a bar showing a grayscale color spectrum running from 0 (white) to 50 (black). We scaled the ratings to lie between 0 and 1 (brightest to darkest). The nouns in this dataset were specifically chosen to represent the entire spectrum of brightness values (e.g., “charcoal” vs. “snow”), and to include concepts for which brightness was a relevant feature. We removed three adjectives from the original dataset to focus solely on nouns (“black,” “white,” and “gray”). The authors also collected ratings for adjective‐modified versions for each noun, which we use in Experiment 2.

The *Binder dataset* 2 consists of semantic ratings collected by Binder et al. (2016). Here, the authors aimed to create a dataset of conceptual feature ratings that was informed by known modalities of neural information processing. The authors settled on 65 semantic features. Participants (*n* = 1743) were asked to rate the relevance of each semantic feature to a word's meaning on a scale from 1 to 6. Each participant was assigned a single word and provided the ratings for all 65 features. As this task was crowdsourced on Amazon Mechanical Turk, the authors included a quality metric to ensure that participants focused on the task. As such, the correlation between a participant's vector and the group average vector of a word was calculated and if this did not exceed a minimum value of *r* = .5, the participant was discarded. The original Binder dataset included two features related to the brightness of a concept: ratings on the degree to which each concept is visually bright or dark. To transform these features into a brightness metric similar to that used in the S&T‐S dataset, we first scaled each dimension between 0 and 1, subtracted the scaled dark dimension from the scaled bright dimension, and then transformed the output to ensure that all values fell between 0 and 1. This way, the spectrum of brightness ratings mimicked Solomon and Thompson‐Schill's, such that stereotypically bright words (e.g., “sun”) had a low rating (0.133), while stereotypically dark words (e.g., “crow”) had high ratings (0.949). It is important to note that Solomon and Thompson‐Schill (2020) specifically selected items for which brightness is a highly relevant property. In contrast, the Binder dataset includes many items which are not strongly associated with a particular level of brightness; hence, many items were clustered around the midpoint of the brightness spectrum. For our second perceptual feature, shape, we also used ratings from the Binder dataset.

We used two versions of the Binder dataset, one which contained only concrete nouns and another that contained both abstract and concrete nouns. The concrete version of the dataset was created by filtering the original dataset on type and super category. We filtered type to only contain items classed as a “thing” or “event,” with super category filtered to include “artifacts,” “living objects,” “natural objects,” and “physical states.” This resulted in a dataset of 274 concrete nouns. For the investigations that included abstract items, we additionally filtered the super category by “abstract entity,” “event,” and “mental entity.” This resulted in a dataset of 433 items, with 275 concrete items and 159 abstract items. We chose to explore the performance of concrete concepts alone as Utsumi (2020) found poor performance for the prediction of perceptual properties for abstract concepts. Therefore, we wanted to test the effect of excluding these.

#### 2.1.2 Embeddings

We used three sets of pretrained word embeddings: Word2Vec, BERTbase, and BERTLarge. This allowed us to make a comparison between word embeddings that can leverage contextual information (contextualized) versus those that are static and independent of context (decontextualized). Moreover, we specifically used pretrained embeddings, rather than training our own, as we wanted to assess the predictive ability of publicly available embeddings that researchers may use to shed light on the conceptual representations of words (Günther, Rinaldi, & Marelli, 2019; Pereira, Gershman, Ritter, & Botvinick, 2016). We also included *one‐hot* vectors as a baseline comparison for each experiment in order to test the influence of our feedforward neural network training. Here, each word is encoded as a vector whose length is equal to the number of words in the vocabulary. For a single word representation, the node associated with that word (e.g., “charcoal”) will be “on,” while all other nodes are switched “off.” As such, one‐hot vectors represent a simple lexical‐coding scheme that contains no semantic information.

*Word2Vec* embeddings are an example of static word embeddings, meaning that they assign a fixed vector to each word, regardless of the context in which the word is being used (Mikolov et al., 2013). We used pretrained embeddings from a Word2Vec model trained on a section of the Google News dataset (∼100 billion words). The dimensionality of the embeddings is *d* = 300, and previous evidence demonstrated that they are a good fit to human semantic judgments across a range of tasks (Pereira et al., 2016). Further details on these pretrained embeddings can be found on the Google Code Archive (https://code.google.com/archive/p/word2vec/).

BERT embeddings are an example of contextualized word embeddings and were extracted from BERT (Bidirectional Encoder Representations from Transformers), a Large Language Model (LLM) based on the Transformer architecture (Devlin et al., 2018). The model is trained on a masked language modeling task, where language samples are provided with 15% of the words masked at random and the model is trained to predict the masked words. We used the HuggingFace Transformers API to access two different pretrained models, which differ in size (bert‐base‐uncased and bert‐large‐uncased) (Wolf et al., 2020). These models were pretrained on two corpora (∼3 billion words), BookCorpus and English Wikipedia, and tokenized using WordPiece, a subword‐based tokenization algorithm. We extracted the corresponding word embedding from the last hidden layer, and if the noun was split into separate subwords, we extracted the subword embeddings and averaged them to represent the entire word (dimensionalities: *BERTbase*: *d* = 768; *BERTLarge*: *d* = 1024). We chose to extract embeddings from the last hidden layer as previous research has demonstrated that semantic features are better represented by higher layers (Jawahar, Sagot, & Seddah, 2019; Turton et al., 2021).

To create our *context‐free* condition, we replicated the method from Turton et al. (2021) for creating a “static” version of BERT embeddings. For each concept, we randomly extracted ∼250 sentences containing the target word from the one Billion Words Benchmark corpus (Chelba et al., 2014) accessed via HuggingFace (https://huggingface.co/datasets/lm1b). To do this, we started with a target word (e.g., “coffee”) and the shuffled train partition of the corpus (*n* = 30,301,028). We then searched through the corpus, using string match to identify sentences that contained our target word, and saved examples of the first 250 sentences that were found. These sentences were then cleaned to remove extraneous punctuation marks and whitespace. For each concept, we ran each of the 250 tokenized corpus sentences through BERT, located the position of the target word in the sentence, and extracted its word‐level embedding (or the averaged subword‐level embedding). We then averaged the 250 embeddings together, which was used as input to our neural network.

To create our *contextually prompted* condition, we generated specific prompts for the nouns depending on the feature to be predicted. For brightness, we initially used “the brightness of [noun].” However, this phrasing could be considered somewhat unusual and unnatural for some concepts in the dataset (e.g., most people would describe crows as being dark in color, not dark in brightness). As such, we also tested a second prompt, “the color of [noun],” which is twice as frequent in the Google n‐grams corpus.^3^ We present results from both prompts and used the best‐performing prompt in comparisons with other embeddings. For predicting shape, we used “the shape of [noun].” We then ran these tokenized phrases through BERT, located the target word at the end of the phrase, and extracted its word‐level embedding (or averaged subword‐level embedding) as input to our neural network. As such, we had three versions of the contextually prompted condition: brightness, color, and shape.

#### 2.1.3 Model

Following a similar approach to other studies (Sommerauer & Fokkens, 2018; Turton et al., 2021, 2020; Utsumi, 2020), we trained a three‐layer feed‐forward neural network to predict human feature ratings from our word embeddings. The model was implemented in PyTorch (Paszke et al., 2019) and consisted of an input layer (dimensions dependent on the input embedding), a ReLU activation function, a dropout layer with *p* = .2, a hidden layer (dimensions dependent on the investigation type), and a single output unit (*d* = 1) with a sigmoid activation function to normalize predictions between 0 and 1. For our training procedure, we used *k*‐fold cross‐validation where *k* = 10. In each fold, models were trained with 90% of concepts and were then tested on their ability to predict the relevant feature for the remaining 10% of concepts. The hyperparameters used for model training were: learning rate = 0.01, bias = –2, momentum = 0.9, and a weight decay = 10^−6^. We optimized using stochastic gradient descent. We also performed hyperparameter tuning for the number of hidden units and number of epochs to train, using gridsearch and nested cross‐validation (*k* = 3). We kept the tuned hyperparameters the same across specific experimental comparisons to ensure fairness.

#### 2.1.4 Evaluation

To reduce random noise, for each investigation, 10 different models were initialized with randomized starting weights. Each of the 10 models was trained and tested according to the 10‐fold cross‐validation scheme described earlier. Differences in model performance across the 10 implementations were small (standard deviations of errors across models are presented in the Supplementary Materials). We obtained a single prediction for each concept by averaging the predictions of the 10 models. We evaluated the performance of the different embeddings using mean squared error (MSE) and *R* ^2^. The MSE was calculated as the average of squared errors between model prediction and the human rating for each concept. The *R* ^2^ for the correlation between the model predictions and the human ratings was calculated by fitting an ordinary least squares regression model. Additionally, we ran statistical tests on comparisons of interest for both brightness and shape, outlined below. To evaluate these comparisons, we used the Wilcoxon signed‐rank test, which is a paired‐samples, nonparametric test, comparing squared errors for each noun. For shape and brightness, we compared:

- Word2Vec versus context‐free BERT
- Context‐free BERT versus contextually prompted BERT

For the brightness experiments, we also compared:

- “Brightness” contextually prompted BERT versus “color” contextually prompted BERT

For experiments using the Binder dataset, we also compared performance for:

- Brightness versus shape

### 2.2 Results

#### 2.2.1 Solomon and Thompson‐Schill dataset

**Fig. 2:**
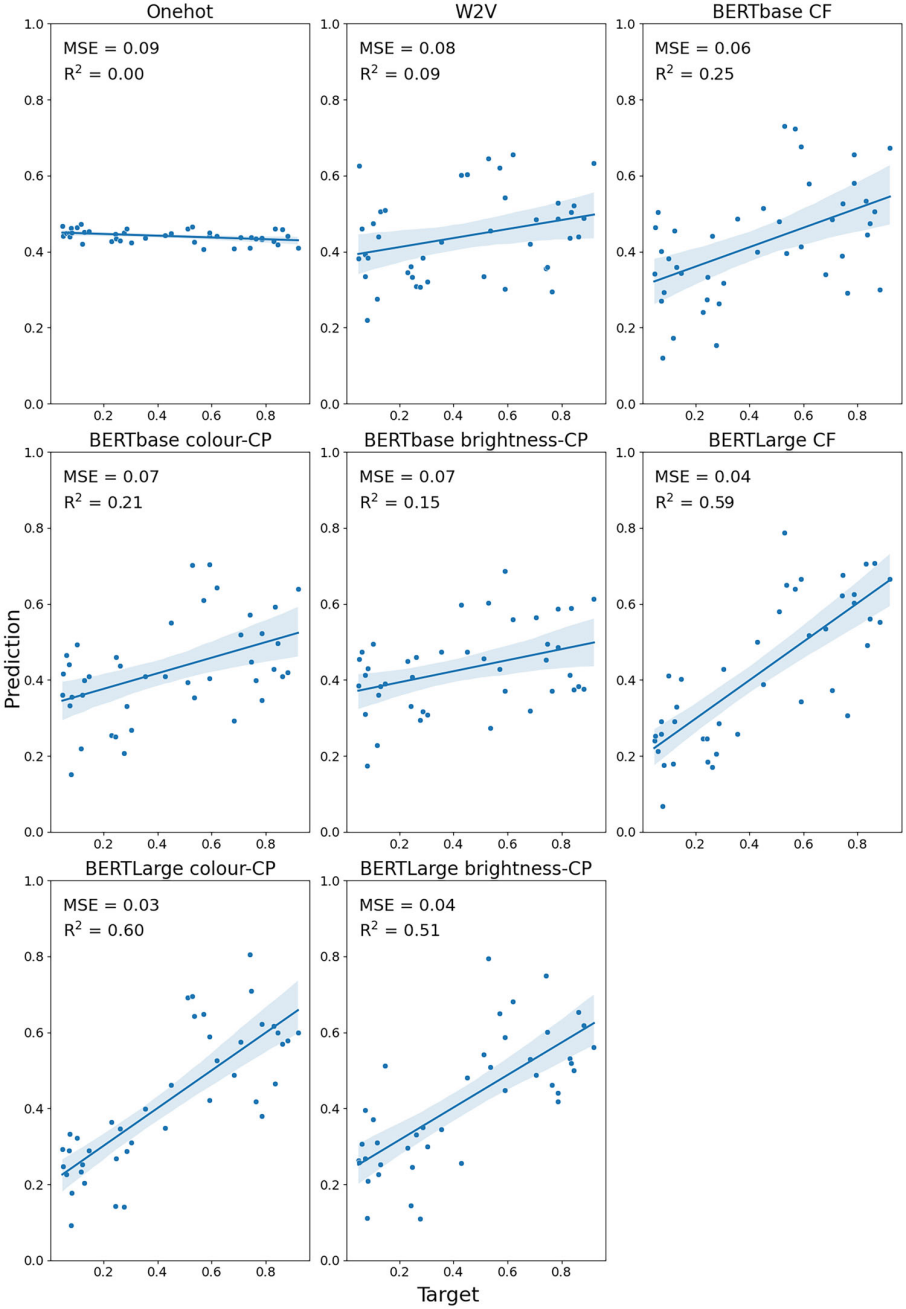
Predicted versus target brightness values for the S&T‐S nouns. Shaded areas indicate 95% confidence interval for regression. CF, context‐free; CP, contextually prompted.

Fig. 2 presents an overview of the noun results for each embedding on the S&T‐S dataset, along with the MSE and *R* ^2^.

As expected, the model trained with one‐hot vectors which contain no semantic information was unable to predict brightness of the target concepts. Predicted brightness values in this model were tightly clustered around 0.5 and were unrelated to the target concept's brightness. Models trained with embeddings performed better. In general, BERTLarge embeddings appeared to have the best performance, with *R* ^2^ values ranging from .5 to .6. Thus, although previous studies have reported poor prediction of brightness/darkness, here we found that embeddings can predict these to a reasonable extent. This may be because we are focusing particularly on a set of nouns for which brightness is a highly relevant feature. Table 1 presents an overview of the averaged performance for each embedding type. The MSE scores and standard deviation for each run of the model can be found in the Supplementary Materials.

**Table 1:** Mean squared error and *R* ^2^ scores for each embedding type on the Solomon noun dataset

	MSE	*R* ^2^
Onehot	0.09	.00
Word2Vec	0.08	.09
BERTbase context‐free	0.06	.25
BERTbase color‐contextually prompted	0.07	.21
BERTbase brightness‐contextually prompted	0.07	.15
BERTLarge context‐free	0.04	.59
BERTLarge color‐contextually prompted	0.03	.60
BERTLarge brightness‐contextually prompted	0.04	.51

For the decontextualized embeddings, we found that the context‐free BERTLarge embeddings performed significantly better than Word2Vec (*p* = 6.97 × 10^−6^). This was also true for the context‐free BERTbase embeddings, but to a lesser extent (*p* = .04). Turning to the contextually prompted embeddings, we found no significant difference between the type of prompt (“the color of” vs. “the brightness of”) for either BERTbase (*p* = .20) or BERTLarge (*p* = .37). As such, we chose to compare the “color” contextual prompt for our comparison with the context‐free embeddings as the *R* ^2^ was higher. We found no significant difference between the contextually prompted and context‐free conditions for either BERTbase (*p* = .33) or BERTLarge (*p* = .66). This suggests the addition of a contextual prompt does not improve the performance for predicting brightness on the S&T‐S dataset.

#### 2.2.2 Binder dataset: Brightness

**Fig. 3:**
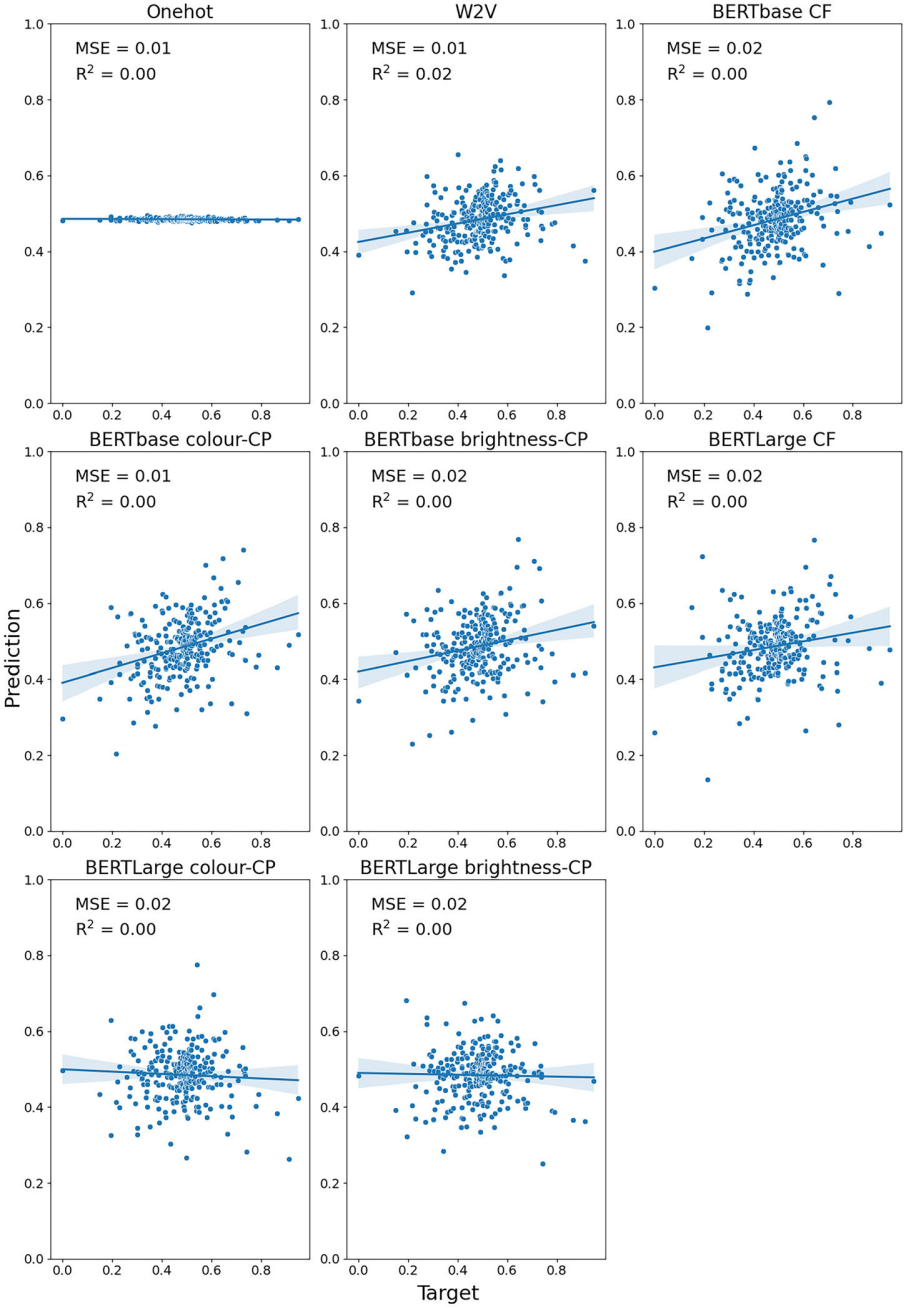
Predicted versus target brightness values for the Binder concrete‐only nouns. Shaded areas indicate 95% confidence interval for regression. CF, context‐free; CP, contextually prompted.

**Fig. 4:**
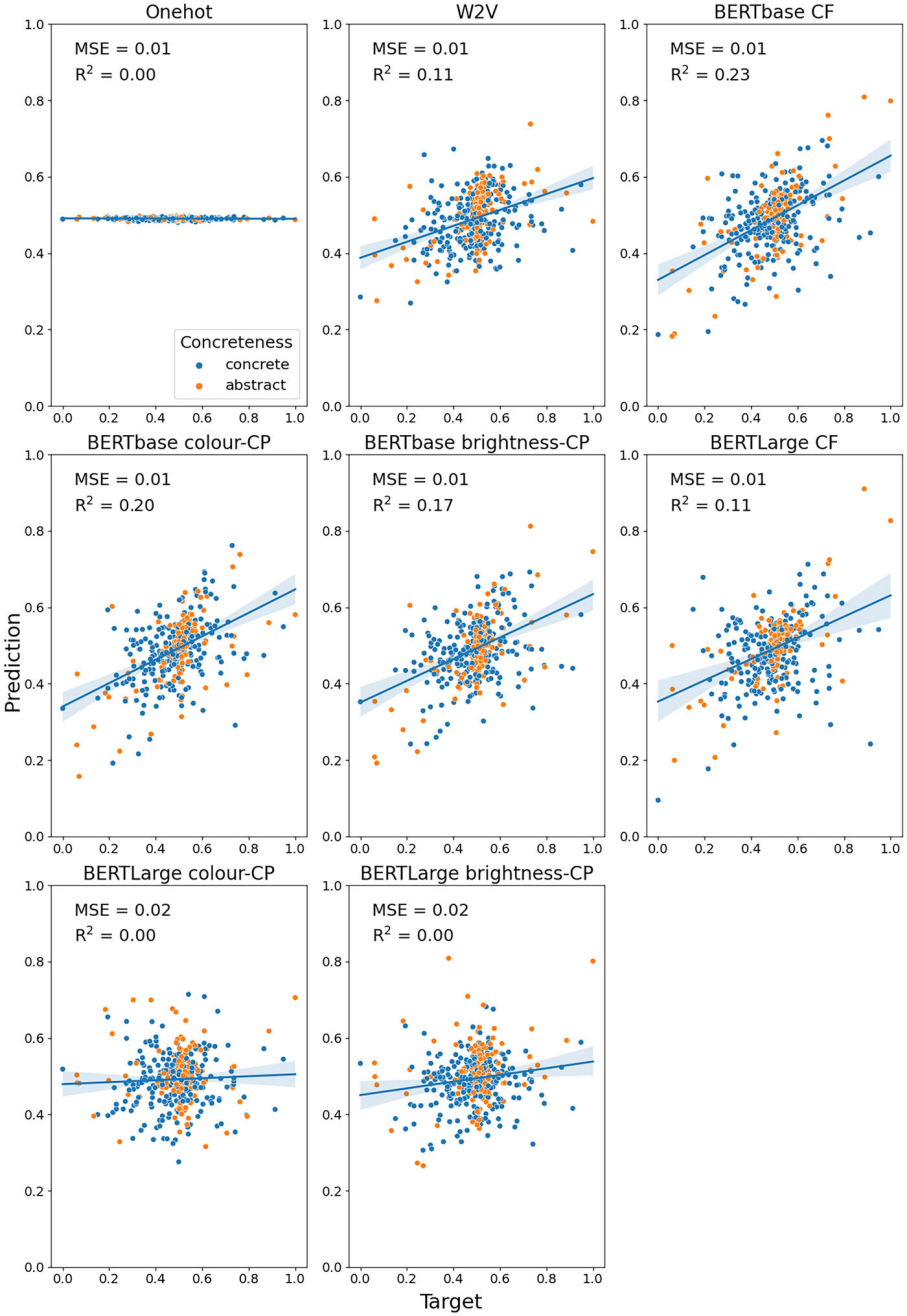
Predicted versus target brightness values for the Binder concrete (blue) and abstract (orange) nouns. Shaded areas indicate 95% confidence interval for regression. CF, context‐free; CP, contextually prompted.

Fig. 3 (concrete nouns only) and Fig. 4 (concrete and abstract nouns) present the MSE and *R* ^2^ for each embedding when the model was trained to predict brightness for the Binder dataset. In general, both dataset configurations produced a similar pattern of results. Overall, the best performing embedding was the context‐free BERTbase condition with both concrete and abstract nouns (MSE = 0.01, *R* ^2^ = .23).

Overall, we found poorer performance across the embedding types when predicting brightness in the Binder dataset compared with the S‐T&S dataset (see Table 2; maximum *R* ^2^ = .23 vs. .60). This suggests that brightness information is not as well represented in the embeddings for this larger dataset that contains many items where brightness is not a highly relevant property.

**Table 2:** Mean squared error and *R* ^2^ scores for each embedding type on the Binder noun datasets predicting brightness

	MSE	*R* ^2^
*Onehot*		
Concrete	0.01	.00
Concrete+abstract	0.01	.01
*Word2Vec*		
Concrete	0.01	.02
Concrete+abstract	0.01	.11
*BERTbase context‐free*		
Concrete	0.02	.00
Concrete+abstract	0.01	.23
*BERTbase color‐contextually prompted*		
Concrete	0.01	.00
Concrete+abstract	0.01	.20
*BERTbase brightness‐contextually prompted*		
Concrete	0.02	.00
Concrete+abstract	0.01	.17
*BERTLarge context‐free*		
Concrete	0.02	.00
Concrete+abstract	0.01	.11
*BERTLarge color‐contextually prompted*		
Concrete	0.02	.00
Concrete+abstract	0.02	.00
*BERTLarge brightness‐contextually prompted*		
Concrete	0.02	.00
Concrete+abstract	0.02	.00

In our comparison of the decontextualized embeddings, we found that the context‐free BERT embeddings outperformed Word2Vec on the concrete+abstract dataset for both BERTbase (*p* = .005) and BERTLarge embeddings (*p* = .05). However, this was not the case for the concrete‐only dataset (base: *p* = .33; large: *p* = .34). This suggests that the improved predictive ability of the context‐free BERT embeddings over Word2Vec may stem from better prediction of abstract concepts in this dataset. For the comparison of the prompt in our contextually prompted conditions, we found that embeddings with the “color” contextual prompt performed better than embeddings with the “brightness” contextual prompt for BERT embeddings in most cases, though this was only significant for the BERTbase concrete‐only dataset (*p* = .01). As such, we used the “color” prompted embeddings for our statistical comparison with the context‐free embeddings. We found a statistical difference between the context‐free and contextually prompted conditions for BERTLarge embeddings (concrete: *p* = .02; concrete+abstract: *p* = 2.48 × 10^−7^), but contrary to expectations, the context‐free embeddings had better prediction performance. Moreover, for the BERTbase versions, we found no statistical difference in prediction performance (concrete: *p* = .09; concrete+abstract: *p* = .96).

#### 2.2.3 Binder dataset: Shape

**Fig. 5:**
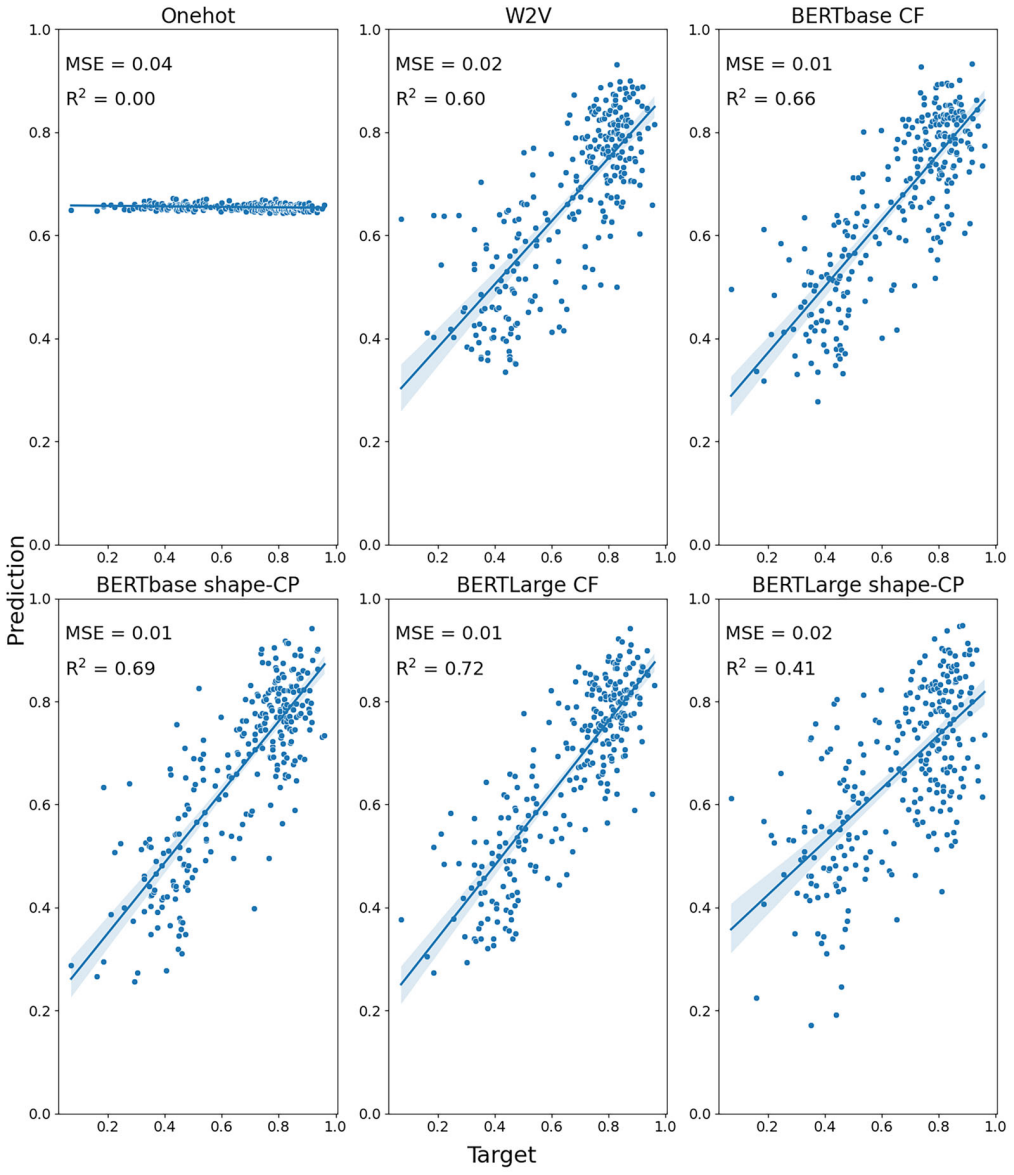
Predicted versus target shape values for the Binder concrete‐only nouns. Shaded areas indicate 95% confidence interval for regression. CF, context‐free; CP, contextually prompted.

**Fig. 6:**
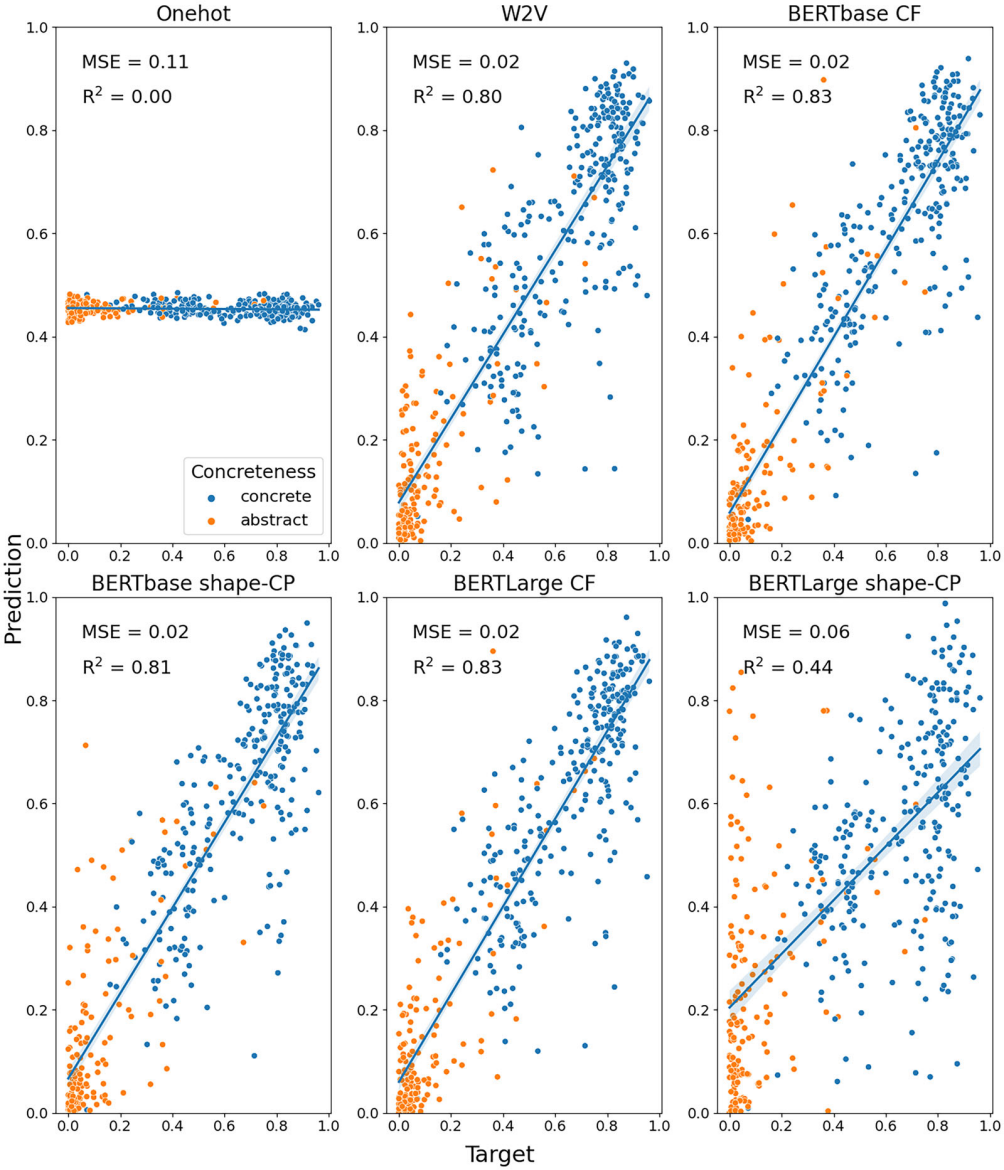
Predicted versus target shape values for the Binder concrete (blue) and abstract (orange) nouns. Shaded areas indicate 95% confidence interval for regression. CF, context‐free; CP, contextually prompted.

Next, we move onto the comparison for predicting the relevance of shape for different concepts. See Fig. 5 (concrete‐only) and Fig. 6 (concrete+abstract) for an overview of the MSE and *R* ^2^ for this investigation.

As expected, based on previous studies, the embeddings led to very good performance in predicting the relevance of shape (Turton et al., 2021; Utsumi, 2020). An overview of embedding performance is presented in Table 3. The best performing embeddings were the context‐free BERT embeddings on the concrete+abstract dataset, with no difference in performance between the base and large versions (base: MSE = 0.02, *R* ^2^ = .83; Large: MSE = 0.02, *R* ^2^ = .83). In general, the addition of abstract nouns improved model performance for most embedding types. This is likely related to the fact that shape values for abstract concepts cluster at the lower end of the spectrum. This strong distinction between concrete and abstract nouns may have helped bootstrap learning of the mapping between nouns and the relevance of shape.

**Table 3:** Mean squared error and *R* ^2^ scores for each embedding type on the Binder noun datasets predicting shape

	MSE	*R* ^2^
*Onehot*		
Concrete	0.04	.00
Concrete+abstract	0.11	.00
*Word2Vec*		
Concrete	0.02	.60
Concrete+abstract	0.02	.80
*BERTbase context‐free*		
Concrete	0.01	.66
Concrete+abstract	0.02	.83
*BERTbase shape‐contextually prompted*		
Concrete	0.01	.69
Concrete+abstract	0.02	.81
*BERTLarge context‐free*		
Concrete	0.01	.72
Concrete+abstract	0.02	.83
*BERTLarge shape‐contextually prompted*		
Concrete	0.02	.41
Concrete+abstract	0.06	.44

Looking at the decontextualized embeddings, we found significant differences between Word2Vec and BERT embeddings for the majority of conditions, with the context‐free BERT embeddings performing slightly better than the Word2Vec embeddings (base: concrete+abstract: *p* = .004; large: concrete: *p* = .002; concrete+abstract: *p* = .02). This was not the case for the BERTbase embeddings on the concrete‐only dataset (*p* = .23). Moreover, in our comparison between context‐free and contextually prompted BERT embeddings, we found that the context‐free BERTLarge embeddings had significantly greater prediction performance than the contextually prompted embeddings (concrete: *p* = 9.64 × 10^−9^; concrete+abstract: *p* = 4.33 × 10^−18^). However, this trend was not apparent for the BERTbase embeddings (concrete: *p* = .21; concrete+abstract: *p* = .06). As such, it appears that the addition of a feature‐specific prompt can lead to worse prediction performance for the perceptual feature of shape.

Finally, we also statistically compared the best‐performing embeddings for brightness and shape features on both dataset configurations. For the concrete‐only dataset, we selected the context‐free BERTbase embedding for brightness and the context‐free BERTLarge embedding for shape. Here, there was no significant difference in performance (*p* = .63). For the concrete and abstract dataset, we chose the context‐free BERTbase embeddings for both features. Here, we found a significant difference between performance, with better prediction performance for shape than for brightness (*p* = .01). This suggests that shape is a better represented perceptual feature in these types of word embeddings than brightness, which is consistent with previous results (Turton et al., 2021, 2020).

### 2.3 Discussion

In Experiment 1, we found good prediction of brightness for the S&T‐S dataset, but not for the Binder dataset, suggesting that even difficult perceptual properties can be predicted when focusing on a subset of nouns where the feature is particularly salient. Replicating previous findings, we also found very good prediction performance for the relevance of shape to concepts (Turton et al., 2021; Utsumi, 2020). In general, we found that context‐free BERT embeddings outperformed Word2Vec embeddings, which aligns with prior findings that aggregated contextualized representations are better predictors of semantic features than static embeddings (Bommasani et al., 2020; Turton et al., 2021). However, contrary to our predictions, we found the contextually prompted embeddings often performed worse than the context‐free embeddings. We consider the reasons for this in the General Discussion. In addition, one‐hot vector representations were entirely unable to predict any perceptual ratings, unlike the pretrained word embeddings. This confirms that success in prediction is a consequence of the semantic information present in the word embeddings and not the neural network we used to map from embeddings to ratings.

## 3 Experiment 2

Until now, contextualized embeddings have primarily been tested on their ability to predict the perceptual properties of nouns. However, how these embeddings represent the perceptual properties of multi‐word expressions, such as adjective–noun phrases, is an important consideration. Linguistic theory states that adjectives modulate the properties of nouns and they frequently do so in a nonuniform manner (Solt, 2019). In particular, the linguistic literature makes a distinction between subsective adjectives whose meaning is context‐sensitive, and, therefore, depends on the comparison class that they modify such as “tall,” and intersective adjectives that have a more context‐insensitive meaning, such as color terms (Demonte, 2019; Partee, 2007). As a subtype of subjective adjectives, relative gradable adjectives such as “slow”/“tall” are additionally characterized by vagueness and have even been theorized to not directly denote properties. Instead, it has been argued that the denotation is only ascribed meaning during the process of composition (Kennedy, 2007, 2012).

Traditional theories on the composition of conceptual combinations include the selective modification model, which specifically focuses on adjective–noun combination. Here, it is assumed that concepts are represented as schema‐like structures with sets of dimensions and corresponding values, similar to prototype theory (Hampton, 2015; Rosch & Mervis, 1975; Rumelhart, 1980). During combination, an adjective's primary feature is reweighted onto the noun concept (Smith, Osherson, Rips, & Keane, 1988; Smith & Osherson, 1984). However, criticisms of the selective modification model note that the process of combination is more complex, especially when expanded to other conceptual combinations such as noun–noun compounds (Hampton, 2015; Murphy, 1988). In contrast, the concept specialization view states that combination occurs through specialization of the head noun concept when one of its “slots” is filled by the modifying concept (Cohen & Murphy, 1984; Murphy, 1988, 2004). The theory emphasizes the role of general background knowledge and reasoning in forming conceptual combinations (Murphy, 2004). In summary, theories on conceptual combination illustrate the notion that the combinatorial process itself is highly idiosyncratic and dependent on the composing concepts (Coutanche, Solomon, & Thompson‐Schill, 2019). This suggests that adjective–noun phrases may be a particular case in which static embeddings are insufficient in capturing the underlying semantics, since the process of combination shifts the representation of both words in an unpredictable fashion.

Adjective–noun phrases are a valuable way to isolate the integration process of conceptual combination as they are independent of additional processes of property selection. Solomon and Thompson‐Schill (2020) demonstrated this by asking people to rate the perceptual feature of brightness for adjective–noun phrases. They found that the adjectives “dark” and “light” modified people's perceptions of the brightness of nouns, but they did not do so in an additive fashion: the adjectives had more of an impact on some nouns than others. For example, for a mid‐brightness noun, such as “paint,” there was a big difference between the perceived brightness of its light (“light paint” = 0.112) and dark (“dark paint” = 0.867) versions. For other nouns with a more extreme and invariant perception of brightness, such as “charcoal,” the adjectives had less of an effect (“light charcoal” = 0.565; “dark charcoal” = 0.930). Examples such as these present as clear test cases where we could ask if contextualized embeddings have an advantage in perceptual prediction, compared to static embeddings, in capturing these complex interactions between adjectives and nouns. As such, for our second experiment, we explored whether contextualized embeddings could accurately predict properties for modified adjective–noun phrases, and whether targeted prompts toward the relevant property improves this behavior.

**Fig. 7:**
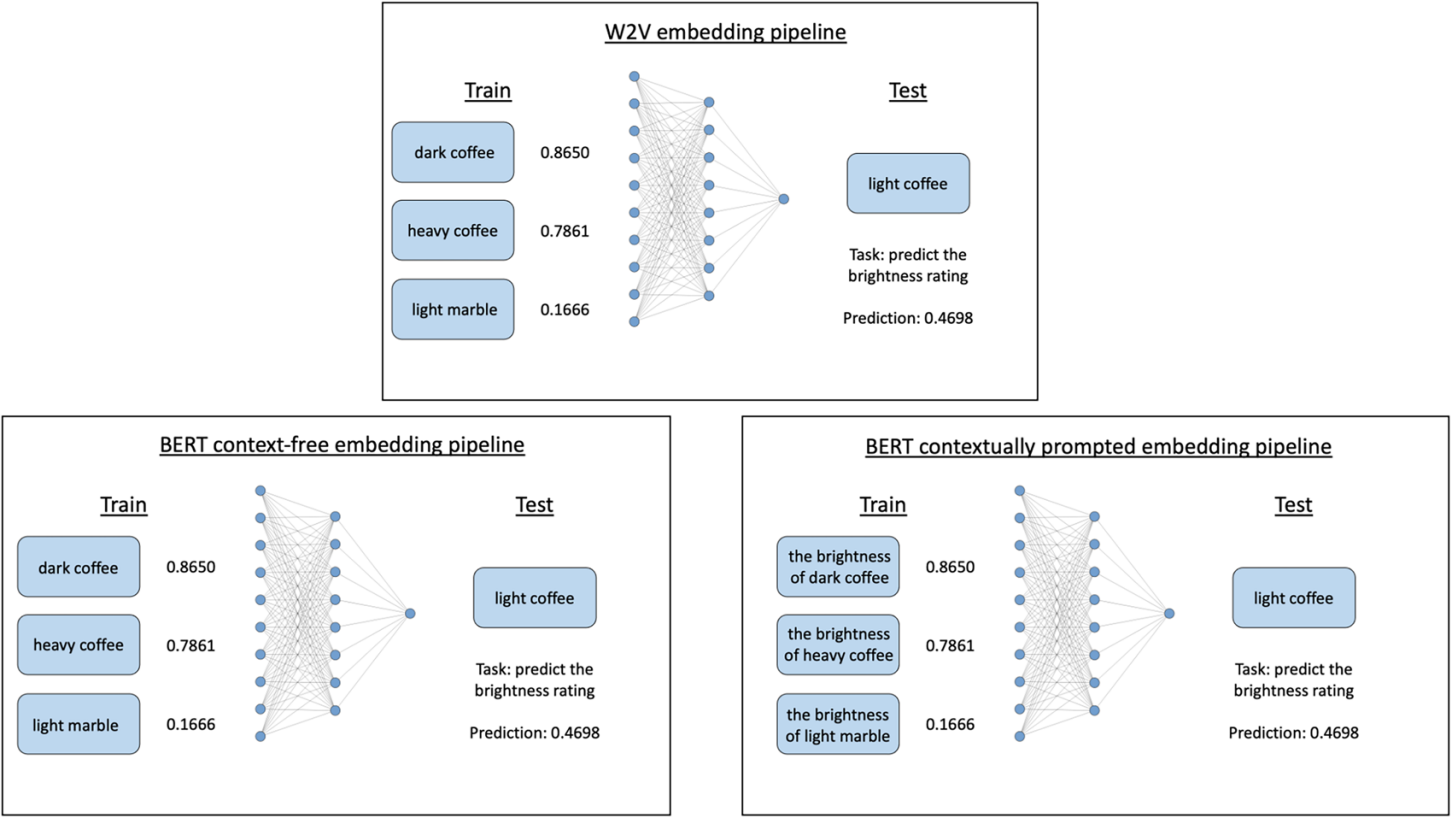
Experiment 2 pipelines.

To test this, we again used Word2Vec embeddings, contextually prompted BERT embeddings, and context‐free BERT embeddings. For the Word2Vec embeddings, we extracted each of the adjective and noun embeddings and concatenated them to represent adjective–noun phrases. As we wished to evaluate predictions for the unmodified noun, alongside the “light” and “dark” versions, we also paired the nouns with an adjective that was uninformative with regard to brightness. We chose “heavy” for this because it is a high‐frequency adjective (similar to “light” and “dark”) that can be applied to objects, while conveying no information about their brightness. Our training paradigm for this investigation tested each model's prediction performance on unseen adjective–noun phrases. For the BERT embeddings, we again compared contextually prompted embeddings with context‐free embeddings. As we were interested in multi‐word expressions, we used BERT's classification (CLS) token to represent the entire phrase (see Methods). For the context‐free conditions, we extracted the CLS token for the adjective–noun phrase alone, while for the contextually prompted conditions, we used the CLS token for the feature‐prompted phrase (e.g., “the brightness/color of dark paint”). Fig. 7 presents an overview of our experiment pipelines for Experiment 2.

### 3.1 Methods

#### 3.1.1 Dataset

We used the S&T‐S dataset for our adjective–noun investigations. For this study, we used all three rating types: the unmodified noun ratings (as used in Experiment 1), and the “dark” and “light” adjective‐modified ratings. These ratings were originally scored between 0 and 50 (bright to dark), which we transformed to between 0 and 1 (bright to dark). As there are 42 nouns in the dataset, there were a total of 126 adjective–noun phrases.

#### 3.1.2 Embeddings

For the *Word2Vec* embeddings, we used the pretrained embeddings described in Experiment 1. We concatenated the adjective and noun embeddings for each phrase (*d* = 600). For the *BERT* embeddings, we used the CLS token, which acts as a combined representation of the adjective and noun, rather than concatenating the word‐level embeddings. The CLS token is a classification token that is required at the beginning of every sentence input into BERT. It is understood as a sentence‐level representation of the input, generated with classification tasks in mind (Devlin et al., 2018; Munikar, Shakya, & Shrestha, 2019). As it constitutes a broad representation of the meaning of multi‐word inputs, we used the CLS token as the entire adjective–noun representation in our analyses. For our contextually prompted condition, we input phrases such as “the brightness of dark charcoal” into BERT and extracted the CLS token for the entire phrase. In contrast, for our context‐free condition, we input only the adjective–noun phrase, for example, “dark charcoal” and extracted the CLS token. This meant that our BERT embeddings always had the same dimensionality (base: *d* = 768; large: *d* = 1024). It was not possible to average context‐free embeddings over multiple sentence contexts (as in Experiment 1) because many of the adjective–noun phrases did not appear in the one Billion Words Benchmark corpus. Finally, we again used a one‐hot encoding scheme to act as a baseline model. Inputs to this model consisted of one unit for each adjective–noun phrase.

#### 3.1.3 Model

We again used 10‐fold cross‐validation to evaluate our models. In Experiment 1, each test fold contained a subset of nouns that the model was not trained on so that we could test generalization to these novel nouns. In the present investigation, we were instead interested in how accurately embeddings could predict the brightness of novel adjective–noun combinations, having been trained on the same adjectives and nouns in different combinations. Accordingly, for each train–test split, we made sure that at least one version of each noun (dark‐noun, light‐noun, or heavy‐noun) appeared in the training set. This ensured that we were not testing on a previously unseen noun, but rather on a previously unseen adjective–noun combination (see Fig. 7 for examples). All other model specifications were the same as our noun investigations, with hyperparameter tuning for the number of hidden units and number of epochs performed specifically for the adjective dataset.

#### 3.1.4 Evaluation

Our evaluation procedures are similar to Experiment 1. We again initialized 10 different models with randomized starting weights to avoid the effect of random noise. Each of the 10 models was trained and tested according to the 10‐fold cross‐validation scheme described for Experiment 1 (see Supplementary Materials for standard deviations of performance across the models). We obtained a single prediction for each adjective–noun combination by averaging predictions from the 10 models. We evaluated the performance of the different embeddings, separated by adjective, using MSE and *R* ^2^. As such, our metrics indexed the ability to predict brightness across the nouns when paired with the same adjective. Additionally, we ran statistical tests on our comparisons of interest, outlined below. We used the Wilcoxon signed‐rank test, which is a paired‐samples, nonparametric test, comparing squared errors for the “light” and “dark” adjective–noun phrases.

- Word2Vec versus context‐free BERT
- “Brightness” contextually prompted BERT versus “color” contextually prompted BERT
- Context‐free BERT versus contextually prompted BERT

The best‐performing embedding out of the prompt comparison (i.e., brightness prompt vs. color prompt) was selected for the context‐free and contextually prompted BERT comparison above. We also present a qualitative evaluation of how well embeddings capture the nonadditive effect of adjective brightness on noun brightness.

### 3.2 Results

**Fig. 8:**
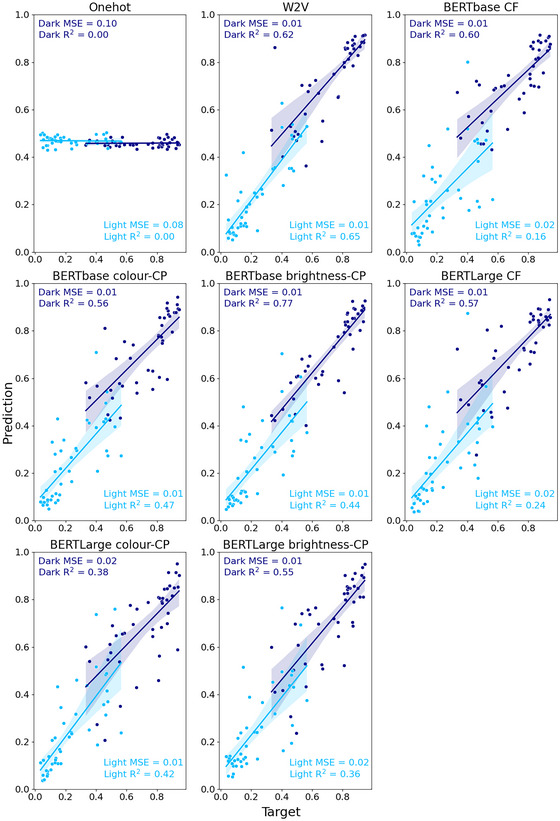
Predicted versus target brightness values for the S&T‐S adjective–noun phrases (dark items = dark blue; light items = light blue). Shaded areas indicate 95% confidence interval for regression. CF, context‐free; CP, contextually prompted.

We evaluate the adjective investigations in a similar way to the noun investigations; however, we report the model performance separated by adjective (see Fig. 8 for an overview).

In general, all of the embeddings could predict the brightness of novel adjective–noun phrases well (see Table 4). We found that the Word2Vec embeddings performed best overall (Dark: MSE = 0.01, *R* ^2^ = .62; Light: MSE = 0.01, *R* ^2^ = .65).

**Table 4:** Mean squared error and *R* ^2^ scores for each embedding type on the Solomon adjective–noun dataset

	MSE	*R* ^2^
*Onehot*		
Dark	0.10	.00
Light	0.08	.00
*Word2Vec*		
Dark	0.01	.62
Light	0.01	.65
*BERTbase context‐free*		
Dark	0.01	.60
Light	0.02	.16
*BERTbase color‐contextually prompted*		
Dark	0.01	.56
Light	0.01	.47
*BERTbase brightness‐contextually prompted*		
Dark	0.01	.77
Light	0.01	.44
*BERTLarge context‐free*		
Dark	0.01	.57
Light	0.02	.24
*BERTLarge color‐contextually prompted*		
Dark	0.02	.38
Light	0.01	.42
*BERTLarge brightness‐contextually prompted*		
Dark	0.01	.55
Light	0.02	.36

Contrary to our predictions, in the analysis of our decontextualized embeddings, we found that Word2Vec embeddings were significantly better at perceptual prediction than the context‐free BERT embeddings (base: *p* = .0001; large: *p* = .0007). For our comparison of prompt effectiveness, we found no significant differences between “the color of” and “the brightness of” prompts (base: *p* = .17; large: *p* = .68). As such, for the contextual analysis, we selected “the brightness of” prompt as it generally had higher *R* ^2^ values. We found that the contextually prompted BERTbase embedding performed significantly better than the context‐free embedding (*p* = .02). However, there was no significant difference between the two for BERTLarge embeddings (*p* = .84).

**Fig. 9:**
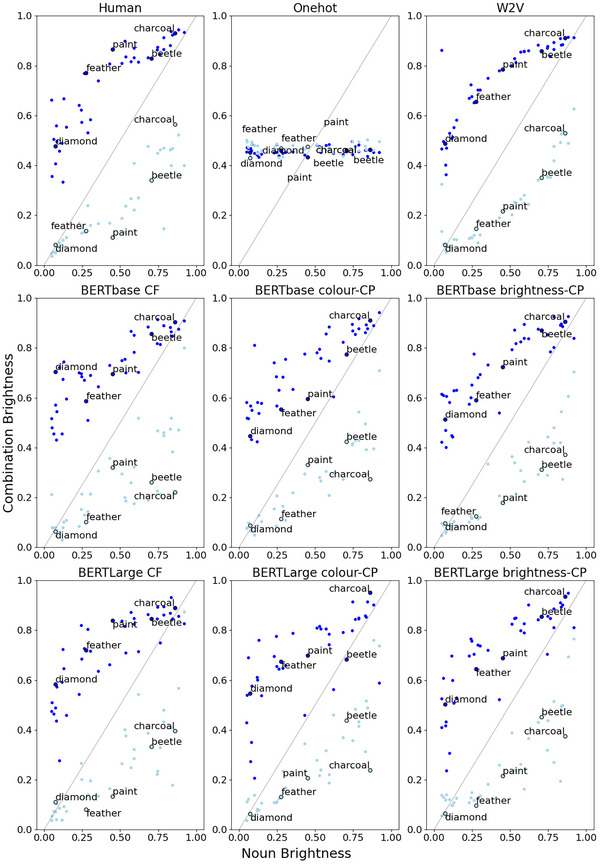
Noun versus combination brightness values for the S&T‐S adjective–noun phrases (dark items = dark blue; light items = light blue) with human ratings (first subplot). CF, context‐free; CP, contextually prompted.

One of the key aspects of the adjective–noun S&T‐S dataset is the nonadditive effect of adjectives on brightness ratings. This “flexible modulation” is shown in the top‐left panel of Fig. 9 which plots the human ratings for adjective–noun brightness (y‐axis) as a function of noun brightness (x‐axis). Adjectives strongly modulate the brightness of nouns that fall in the middle of the spectrum (e.g., “paint”), while they have less effect on nouns with more extreme brightness values (e.g., “charcoal”). We can see a similar pattern (curvature of datapoints) for the word embeddings.

### 3.3 Discussion

Overall, we observed good performance for perceptual prediction on unseen adjective–noun combinations for the pretrained word embeddings, but not for the one‐hot baseline. We also found that embeddings mimic the nonadditive “flexible modulation” evident from human data, which suggests that LLMs can capture complex aspects of conceptual combination. However, here we found that Word2Vec embeddings outperformed context‐free BERT embeddings, in contrast to our Experiment 1 findings. It is possible this is because the adjective and noun are represented separately in our Word2Vec inputs, as these were a concatenation of the two constituent embeddings, whereas our context‐free BERT embeddings are a single, blended representation of both words. We found that the addition of context had a limited impact on perceptual performance, with modest evidence that including a prompt toward the feature could uncover additional feature‐related information.

## 4 General discussion

In this study, we investigated the ability of embeddings from Distributional Semantic Models and Large Language Models to represent perceptual information, comparing embeddings with different levels of contextual constraint. In Experiment 1, we compared contextualized and decontextualized word embeddings on their performance on perceptual prediction for the brightness and shape associated with nouns. In general, we found very good prediction of shape and more modest prediction of brightness, suggesting that language models do capture some perceptual aspects of meaning through exposure to linguistic information alone. Shape was generally better predicted than brightness, replicating previous findings (Chersoni et al., 2021; Turton et al., 2021, 2020). In our novel investigation of context, we found no advantage for contextualizing the embedding with the desired perceptual feature (e.g., “the brightness of charcoal”). In fact, this most often led to worse performance. For Experiment 2, we explored whether word embeddings flexibly represent the modulation of perceptual properties that occurs when nouns are modified by adjectives (e.g., “dark charcoal”). This work extends previous findings to focus on how conceptual features from multiple inputs interact in multi‐word expressions. Overall, the embeddings were successful in predicting the properties of novel adjective–noun combinations, and we found limited evidence for the effectiveness of contextual prompting on perceptual feature prediction. We also found that the nonadditive effect of adjectives on noun brightness was represented within the embeddings, which mimics a key qualitative characteristic of the human dataset (Solomon & Thompson‐Schill, 2020). These results have implications for understanding the degree to which aspects of embodied perceptual experience are coded in the statistics of language.

We found generally good prediction of perceptual features from the different sets of pretrained word embeddings tested. This was even the case for brightness prediction, which is a feature that previous studies found to be poorly predicted (Chersoni et al., 2021; Turton et al., 2021; Utsumi, 2020). One caveat to note is the difference in performance when predicting brightness for the two datasets. Our results suggest that performance may depend critically on the set of concepts used to test predictions. Previous studies relied heavily on the Binder dataset, which covers a wide range of concepts. We found better performance for brightness predictions when we used the S&T‐S dataset, which contains a more tailored set of concepts for which brightness is a relevant feature. Solomon and Thompson‐Schill (2020) curated their dataset to include concepts that represented the spectrum of brightness values. We found that this resulted in better prediction performance in contrast to the Binder dataset, which included many concepts where brightness was not a salient feature. As such, it may be important to use data that cover the spectrum of perceptual feature ratings when assessing the representational ability of word embeddings.

In addition, our findings suggest that shape is more strongly represented in word embeddings than the brightness of a concept, replicating previous results (Chersoni et al., 2021; Turton et al., 2021, 2020). This could be in part because the degree to which a concept has a shape is strongly related to the degree to which the concept is concrete or abstract. More abstract concepts (e.g., “government”) reliably have lower ratings compared to more concrete concepts (e.g., “table”; see Fig. 6). This relationship does not hold for brightness, where some abstract concepts (e.g., “summer”) can be rated highly and many concrete concepts are neutral. As such, it is possible that shape prediction was aided by this feature's correlation with concreteness, which is highly salient both psychologically and linguistically (Barsalou, Dutriaux, & Scheepers, 2018; Paivio, 1990). However, better performance does not seem to stem simply from an effect of bootstrapping from concreteness information, as we observed better prediction performance for shape even when only concrete nouns were included.

In terms of the differences between word embeddings, we found that the context‐free BERT embeddings, which were averaged across many contexts, generally outperformed Word2Vec embeddings in predicting perceptual ratings in Experiment 1. This replicates a previous finding and suggests that probing word embeddings across multiple contexts can lead to more robust representations than static embeddings (Bommasani et al., 2020; Turton et al., 2021; Vulić et al., 2020). From our novel investigations with the addition of a contextual prompt, we found that the contextually prompted BERT embeddings did not result in better performance than our context‐free BERT embeddings. In some circumstances, we actually found worse performance. This does not align with what we observe in humans, where context has a powerful effect in shaping how particular concepts are retrieved and interpreted (see Yee & Thompson‐Schill, 2016 for a comprehensive review). In one apposite example, Bermeitinger, Wentura, and Frings (2011) gave participants a task which drew attention to the shape of concepts, interspersed with a semantic priming task. This resulted in greater priming for concepts where shape was a relevant feature, compared to less shape‐relevant concepts. This type of semantic facilitation based on the interaction between the context and a concept's features is a robust finding using both behavioral and neuroimaging methods (Hoenig, Sim, Bochev, Herrnberger, & Kiefer, 2008; Hoffman, McClelland, Ralph, & M., 2018; Kuhnke, Kiefer, & Hartwigsen, 2020; Tabossi & Johnson‐Laird, 1980; Van Dam, Rueschemeyer, Lindemann, & Bekkering, 2010; van Dam et al., 2012; Yee, Ahmed, & Thompson‐Schill, 2012). Findings like these indicate that the perceptual features humans activate upon processing a concept are strongly influenced by the recent context. However, contextual prompting does not appear to shape the expression of feature‐specific information in Transformer‐based LLMs in the same way.

Our results suggest that specific perceptual prompts may not engineer representations that better reflect that particular property and that context‐free embeddings (aggregated over many contexts) are more effective. This may be one way in which the semantic representations extracted from language models differ from our understanding of semantic representations in the human brain. Future work that considers a wider range of features and prompts would give greater insights into the ways in which context influences the representation of different semantic features in word embeddings. It could also be possible that newer, larger LLMs are better able to capture context than BERT, which is not as well‐suited to this type of prompting. As such, future explorations of how different model architectures handle this task may also be of interest. Between the two versions of the BERT model, it is interesting to note that BERTLarge did not consistently outperform BERTbase embeddings. BERTLarge is a substantially larger model, with more than three times as many parameters as BERTbase, and was reported to outperform BERTbase on a range of language processing tasks (Devlin et al., 2018). However, others have suggested that the BERT models were significantly undertrained (Liu et al., 2019) and it is possible that with greater training, BERTLarge would have consistently overtaken the smaller model in our perceptual comparisons.

In Experiment 2, we explored the ability to predict perceptual feature values from word embeddings for novel adjective–noun phrases, having had experience of each of their constituents. We found that the Word2Vec embeddings generally outperformed the context‐free BERT embeddings. Here, our Word2Vec embeddings were a concatenation of the adjective and noun embeddings, while for our context‐free BERT embeddings, we took the CLS token to represent the entire adjective–noun phrase. It is possible that having distinct representations of the adjective and noun resulted in the better performance for Word2Vec embeddings in this task. Nevertheless, the BERT embeddings did perform well on this task, which suggests that an integrated phrase‐level representation (i.e., CLS token) also carries perceptual information. We also found a limited impact of contextual prompting on predictions, as our contextually prompted BERTbase embeddings performed significantly better than our context‐free BERTbase embeddings. However, this relationship did not hold for the BERTLarge embeddings. We take this as modest evidence that the provision of a contextual prompt can uncover further feature‐related information. We also found evidence of the nonadditive nature of adjective–noun brightness reflected in the embeddings. This characteristic refers to the variable way in which modification with an adjective impacts the brightness ratings of nouns (Solomon & Thompson‐Schill, 2020). For example, a mid‐brightness concept, such as “paint,” has greater variation in brightness ratings across adjective combinations, than an extreme‐brightness concept, such as “charcoal.” This finding suggests that the type of flexible modulation found in how humans represent concepts is also reflected in word embeddings.

Overall, our study converges with previous investigations in suggesting that a surprising degree of perceptual information can be transferred through linguistic content alone. Utsumi (2020) suggests that language is a realization of experiences in the real world, arguing that the type of statistics used by language models implicitly involve conceptual knowledge from direct experience. Our findings support this view, which is most compatible with a unified account of cognition, where processing integrates embodied and symbolic elements (Andrews et al., 2014; 2009; Louwerse, 2011, 2018). Accounts such as the Symbol Interdependency Hypothesis emphasize the use of both symbol‐to‐symbol mappings, as well as symbol‐to‐world mappings (Louwerse, 2011). The current work adds to the body of literature indicating that semantic representations learnt using distributional methods frequently align with those based on reports of perceptual experience (Chersoni et al., 2021; Lucy & Gauthier, 2017; Sommerauer & Fokkens, 2018; Utsumi, 2020). Additional evidence for this sort of dual representational system is reviewed by Bi (2021), who highlights behavioral and neuroimaging studies of color knowledge on both those with visual experience and those without (i.e., congenitally blind participants). Evidence from neural decoding demonstrated a nonsensory, language‐derived system of color knowledge in both sets of participants, with an additional sensory‐derived representation present for those with visual experience. This suggests that humans can and do acquire perceptual knowledge through language to some degree. This evidence is more in line with a weak embodiment approach, such as that put forward by Dove (2014), who argues that language itself is embodied and interacts with other embodied systems (i.e., perception and action). Another relevant account is the Linguistic and Situated Simulation theory (Barsalou et al., 2008; Santos, Chaigneau, Simmons, & Barsalou, 2011), which proposes that lexical–semantic processing involves the early activation of linguistic information and is supported by later, embodied processes that simulate relevant sensorimotor experiences.

Possible directions for future research include investigating how different types of semantic features are encoded in language for both concrete and abstract concepts. Specifically focusing on meaning within LLMs, Piantadosi and Hill (2022) argued that meaning is not just borne out of grounding, but rather in how concepts relate to each other. They claim that this mapping of interactions between concepts is common to both humans and machines. Exploring the mechanisms behind how LLMs gain this kind of representational ability would be an interesting direction for future work. Another possible direction is to investigate the extent to which perceptual properties of interactions are represented in models. While we have explored the nature of adjective and noun interactions, there are many interactions involved in a myriad of conceptual combinations, such as noun–noun compounds, which warrant further investigation (Coutanche et al., 2019). Moreover, work that considers different implementations in the extraction and testing of word embeddings would address one of the limitations of the current work. For example, this could include different methods for extracting the word embeddings, or the use of more elaborate prompts when contextualizing toward a specific feature. Further research in this area would allow for greater analysis of the generalizability of the current findings.

In conclusion, our current work adds to the recent literature that probes the relationship between word embeddings and human semantic ratings. We replicated previous results that demonstrated generally good performance for the prediction of some perceptual features, namely, shape, while brightness was less well represented. In our novel contributions, we found that the addition of a contextual prompt had limited improvements on the representational ability of word embeddings for perceptual prediction. Moreover, word embeddings do reflect some of the flexible modulation of perceptual features that occurs in semantic compositions, in particular, modification with an adjective. Future research could focus on generating more specific context prompts and extending this to other features. This may reveal further insights into how linguistic context impacts engineered semantic representations.

## Supporting information



Table S1. Standard deviation across MSE scores for each run (10) of model on Solomon noun dataset.
Table S2. Standard deviation across MSE scores for each run (10) of model on Binder dataset predicting brightness.
Table S3. Standard deviation across MSE scores for each run (10) of model on Binder dataset predicting shape.
Table S4. Standard deviation across MSE scores for each run (10) of model on Solomon adjective‐noun dataset.

## Notes

1 The S&T‐S dataset can be accessed here: https://osf.io/7uwn9/

2 The Binder dataset can be accessed here: https://www.neuro.mcw.edu/index.php/resources/brain‐based‐semantic‐representations/

3
https://books.google.com/ngrams/graph?content=the+colour+of%2C+the+brightness+of&year_start=2000&year_end=2019&corpus=en‐2019&smoothing=3&case_insensitive=true
