# Supplementary material for: Leveraging Context for Perceptual Prediction Using Word Embeddings
Source: Cogn Sci. 2025 Jun 7;49(6):e70072. doi: 10.1111/cogs.70072 (PMC12145134; doi:10.1111/cogs.70072)
Supplement: Supplementary file 1 — Table S1. Standard deviation across MSE scores for each run (10) of model on Solomon noun dataset. Table S2. Standard deviation across MSE scores for each run (10) of model on Binder dataset predicting brightness. Table S3. Standard deviation across MSE scores for each run (10) of model on Binder dataset predicting shape. Table S4. Standard deviation across MSE scores for each run (10) of model on Solomon adjective‐noun dataset. [file COGS-49-e70072-s001.docx]

|  | MSE | Standard deviation (σ) |
| --- | --- | --- |
| *Onehot* | 0.09 | 0.002 |
| *Word2Vec* | 0.08 | 0.002 |
| *BERTbase context-free* | 0.06 | 0.002 |
| *BERTbase colour-contextually prompted* | 0.06 | 0.002 |
| *BERTbase brightness-contextually prompted* | 0.07 | 0.003 |
| *BERTLarge context-free* | 0.04 | 0.001 |
| *BERTLarge colour-contextually prompted* | 0.03 | 0.001 |
| *BERTLarge brightness-contextually prompted* | 0.04 | 0.002 |

Table S1. Standard deviation across MSE scores for each run (10) of model on Solomon noun dataset.

Table S2. Standard deviation across MSE scores for each run (10) of model on Binder dataset predicting brightness.

|  | MSE | Standard deviation (σ) |
| --- | --- | --- |
| *Onehot* | | |
| Concrete | 0.01 | 8.71520102225854e-05 |
| Concrete+abstract | 0.01 | 4.3093925851674064e-05 |
| *Word2Vec* | | |
| Concrete | 0.01 | 0.0004 |
| Concrete+abstract | 0.01 | 0.0002 |
| *BERTbase context-free* | | |
| Concrete | 0.02 | 0.0002 |
| Concrete+abstract | 0.01 | 0.0002 |
| *BERTbase colour-contextually prompted* | | |
| Concrete | 0.01 | 0.0004 |
| Concrete+abstract | 0.01 | 0.0004 |
| *BERTbase brightness-contextually prompted* | | |
| Concrete | 0.02 | 0.0003 |
| Concrete+abstract | 0.01 | 0.0003 |
| *BERTLarge context-free* | | |
| Concrete | 0.02 | 0.0002 |
| Concrete+abstract | 0.01 | 0.0002 |
| *BERTLarge colour-contextually prompted* | | |
| Concrete | 0.02 | 0.0002 |
| Concrete+abstract | 0.02 | 0.0002 |
| *BERTLarge brightness-contextually prompted* | | |
| Concrete | 0.02 | 0.0003 |
| Concrete+abstract | 0.02 | 0.0003 |

Table S3. Standard deviation across MSE scores for each run (10) of model on Binder dataset predicting shape.

|  | MSE | Standard deviation (σ) |
| --- | --- | --- |
| *Onehot* | | |
| Concrete | 0.04 | 0.0002 |
| Concrete+abstract | 0.11 | 0.0007 |
| *Word2Vec* | | |
| Concrete | 0.02 | 0.0003 |
| Concrete+abstract | 0.02 | 0.0003 |
| *BERTbase context-free* | | |
| Concrete | 0.01 | 0.0002 |
| Concrete+abstract | 0.02 | 0.0004 |
| *BERTbase contextually prompted* | | |
| Concrete | 0.01 | 0.0004 |
| Concrete+abstract | 0.02 | 0.0003 |
| *BERTLarge context-free* | | |
| Concrete | 0.01 | 0.0001 |
| Concrete+abstract | 0.02 | 0.0003 |
| *BERTLarge contextually prompted* | | |
| Concrete | 0.02 | 0.0004 |
| Concrete+abstract | 0.06 | 0.002 |

Table S4. Standard deviation across MSE scores for each run (10) of model on Solomon adjective-noun dataset.

|  | MSE | Standard deviation (σ) |
| --- | --- | --- |
| *Onehot* | | |
| Dark | 0.10 | 0.01 |
| Light | 0.08 | 0.01 |
| *Word2Vec* | | |
| Dark | 0.01 | 0.006 |
| Light | 0.01 | 0.002 |
| *BERTbase context-free* | | |
| Dark | 0.01 | 0.003 |
| Light | 0.02 | 0.007 |
| *BERTbase colour-contextually prompted* | | |
| Dark | 0.01 | 0.0007 |
| Light | 0.01 | 0.0009 |
| *BERTbase brightness-contextually prompted* | | |
| Dark | 0.01 | 0.0005 |
| Light | 0.01 | 0.001 |
| *BERTLarge context-free* | | |
| Dark | 0.01 | 0.004 |
| Light | 0.02 | 0.005 |
| *BERTLarge colour-contextually prompted* | | |
| Dark | 0.02 | 0.001 |
| Light | 0.01 | 0.001 |
| *BERTLarge brightness-contextually prompted* | | |
| Dark | 0.01 | 0.001 |
| Light | 0.02 | 0.0004 |
